# Assessment of the Status of Patients with Parkinson’s Disease Using Neural Networks and Mobile Phone Sensors

**DOI:** 10.3390/diagnostics10040214

**Published:** 2020-04-12

**Authors:** Yulia Shichkina, Elizaveta Stanevich, Yulia Irishina

**Affiliations:** 1 St. Petersburg State Electrotechnical University “LETI”, 197376 St. Petersburg, Russia; liza_soft_1313@mail.ru; 2 N.P. Bechtereva Institute of the Human Brain of the Russian Academy of Sciences, 197376 St. Petersburg, Russia; irishina@mail.ru

**Keywords:** Parkinson’s disease, recurrent neural network, smartphone, motion sensor, monitoring the condition of patients

## Abstract

Parkinson’s disease (PD) is one of the most common chronic neurological diseases and one of the significant causes of disability for middle-aged and elderly people. Monitoring the patient’s condition and its compliance is the key to the success of the correction of the main clinical manifestations of PD, including the almost inevitable modification of the clinical picture of the disease against the background of prolonged dopaminergic therapy. In this article, we proposed an approach to assessing the condition of patients with PD using deep recurrent neural networks, trained on data measured using mobile phones. The data was received in two modes: background (data from the phone’s sensors) and interactive (data directly entered by the user). For the classification of the patient’s condition, we built various models of the neural network. Testing of these models showed that the most efficient was a recurrent network with two layers. The results of the experiment show that with a sufficient amount of the training sample, it is possible to build a neural network that determines the condition of the patient according to the data from the mobile phone sensors with a high probability.

## 1. Introduction

Parkinson’s disease (PD) is one of the most common neurodegenerative disorders, only second after Alzheimer’s disease. It is a chronic progressive neurodegenerative disease, associated mainly with dopamine deficiency in the subcortical ganglia of the brain and manifests primarily by movement disorders in the form of Parkinson’s syndrome (hypokinesia, or slow movement combined with increased muscle tone of the limbs and trunk, or rigidity and/or tremor), as well as a wide range of non-motor manifestations. A feature of the development of PD is that the neurodegenerative process begins 10 years or more before the appearance of motor disorders, but the diagnosis of PD is possible only with the appearance of the latter. There are no laboratory or instrumental methods that can confirm the diagnosis of PD. Due to neurotransmitter replacement therapy for PD, after a few years, in most cases, the clinical picture of motor disorders is modified, which is manifested by various fluctuations in motor symptoms such as a change in the severity of symptoms during the day depending on dopaminergic therapy, as well as various violent movements (dyskinesias). Due to the progression of the disease (a decrease in the number of dopamine-producing neurons), treatment regimens in the late stages of the disease become more complex and fluctuations and dyskinesias become more pronounced [1,2].

Modern means of observing patients are limited, requiring the time of a doctor and a patient, which limits the frequency of clinical evaluations. Another way to monitor the patient’s condition is to constantly fill a patient diaries. However, the information in diaries is subjective, which leads to significant changes in indicators depending on the mental state of the patient. Consequently, the results of the analysis based on diaries have low accuracy. For example, a study [3,4] showed that only in 11% of cases the patient’s perception corresponds to clinical indicators.

Mobile phones provide automatic, convenient monitoring and recording of observations in real time, which can be invaluable for large-scale studies and personal monitoring of patient health. Patients can simply download the application to their smartphone, which allows the system to collect and analyze data. Developed specialized tests on mobile phones can give medical staff access to long-term measurements of the severity of symptoms and their variations.

The analysis of data obtained using smartphones is extremely difficult due to the large number of diverse types of data selected over long periods of time. The main unresolved tasks for today are: how to simultaneously analyze a wide range of symptoms associated with PD; how to best aggregate and analyze huge volumes of clinically relevant data; in what form should the analysis results be displayed. To solve such problems, it is possible to use neural networks. Research results [5] show that the use of machine learning methods can give a level of confidence in assessing a patient’s condition comparable to a doctor.

This article describes our studies to assess the condition of a patient with Parkinson’s disease based on data from mobile phones on the nature of the use of the phone, such as the angle of rotation and tilt of the phone. The article is organized as follows: the second section provides an overview of existing solutions in the field of monitoring the status of patients with Parkinson’s disease and the distinctive features of our study; the third section provides a brief description of the data collection system and the statement of the problem; the fourth section is devoted to the analysis of possible neural network architectures used to classify the status of patients with Parkinson’s disease and to describe the learning outcomes of these networks; the fifth section describes further research; the sixth and seventh sections discuss the limitations in this study and the study conclusions, respectively.

## 2. Overview of Related Research

The analysis of studies on the use of neural networks to monitor the status of patients with Parkinson’s disease has shown that conditionally all studies can be divided into the following classes:

1. The use of neural networks for predicting Parkinson’s disease in relation to individual parameters of the patient’s condition. These include studies [6,7,8,9]. These studies describe various models of neural networks, as well as interesting and useful results on the simultaneous use of various types of neural networks. The main drawback of these studies is the use of neural networks only of single parameters, such as voice or tremor of limbs or others.
2. The integrated use of artificial intelligence methods for the diagnosis of Parkinson’s disease. For example, studies have been conducted on the use of: A probabilistic neural network (PNN) and classification tree (ClT) [10]; Gaussian models, principal component analysis methods, linear discriminant analysis, least squares support vector method (LS-SVM), probabilistic neural network (PNN), and common neural network regression (GRNN) [11]; methods based on k- clustering medium (KMCFW) and the complex-valued artificial neural network (CVANN) [12]; combination minimum redundancy maximum relevance (mRMR) attribute selection algorithm and CVANN [13]; neural networks and machine learning methods [14]; the wavelet transforms and neural networks [15]; or radial basis function neural network (RBFNN), based on the particle swarm optimization (PSO) and principal component analysis (PCA) [16,17].
3. Comparisons between the effectiveness of the applications of artificial intelligence methods for the diagnosis of Parkinson’s disease, for example, incremental search (IS), Monte Carlo search (MCS), and hybrid search (HS) [18].

It is also possible to classify existing studies by a set of parameters regarding the status of patients with Parkinson’s disease, for which Parkinson’s disease was diagnosed or predicted by:

4. tremor of human limbs [10];
5. voice [12,13,18];
6. genetic factors [14];
7. motor activity [15,19];
8. the results of medical devices, for example, electroencephalogram signals [9].

In all studies, the following equipment was mainly used for data collection:

9. the use of special sensors [15,19,20];
10. the use of mobile phones [21,22];
11. the use of special medical equipment [9,23].

It should be noted that most often, special sensors are used in research; for example, a set of accelerometers and gyroscopes are placed on the body of subjects when they perform a series of standard motor tasks [20]. However, such an approach to measuring the parameters of the condition of patients cannot be applied for everyday monitoring. In contrast to the authors of [15,19,20], we use tools that are more accessible to many patients to measure hand movements. However, in this case we cannot take into account the correlation between the accelerometers on the arm, trunk, and leg. Using the phone, we can, however, collect other important parameters about the condition of patients, such as evaluating memory, attention, voice parameters, emotional state, and others. This is another distinguishing feature of our research, i.e., assessment of the patient’s condition by a large set of parameters.

## 3. Statement of the Problem

Monitoring the patient’s condition is the key to the success of the correction of the main clinical manifestations of PD, including the almost inevitable modification of the clinical picture of the disease against the background of prolonged dopaminergic therapy.

Modern smartphones have built in accelerometers which promise to enable quantifying minute-by-minute activity of patients (e.g., walk or sit) [24]. The quality of smartphones makes it possible to improve medical diagnostics and monitor the patient’s condition.

The data used in this study was obtained using a mobile application in which patients and healthy people solve a wide range of tests that have been agreed by medical staff. When passing the tests, the system evaluates speech, hand tremors, tapping of fingers, speed, balance, and reaction time.

**Figure 1:**
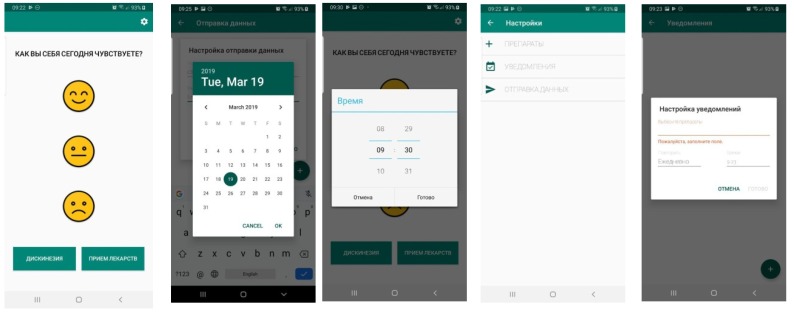
Examples of some patient application windows.

Examples of application windows that the patient interacts with are shown in Figure 1.

In previous articles [22], we described the architecture of our monitoring system for a patient with Parkinson’s disease. The collected indicators are presented in Table 1. Our study involved 28 people, of which 10 patients aged 45 to 80 years with a diagnosis of PD. All subjects gave their informed consent for inclusion before they participated in the study. The study was conducted in accordance with the Declaration of Helsinki, and the protocol was approved at the meeting of the Academic Council of the N.P.Bechtereva Institute of the Human Brain of the Russian Academy of Sciences dated 17 September 2015. (No. 29). Data collection was carried out for one month. As a result, we made 100,000 records on the angles of rotation and tilt of the mobile phone and 5000 records according to the test results.

To train the network, we needed data on how patients felt. Therefore, subjects entered data throughout the day after passing the tests i.e., the time of taking the medicine, the severity of dyskinesia, and assessing their condition. A numerical score was calculated after the completion of its passage according to the results of each test. Then the obtained results were analyzed together with the data entered manually. We data mined the results of the tests and data from device sensors to create a “PD score”, which characterizes the severity of the disease.

The aim of the current study was to build a system for monitoring the status of a patient with Parkinson’s disease based on a set of parameters that can be estimated based on data obtained using mobile phones.

**Figure 2:**
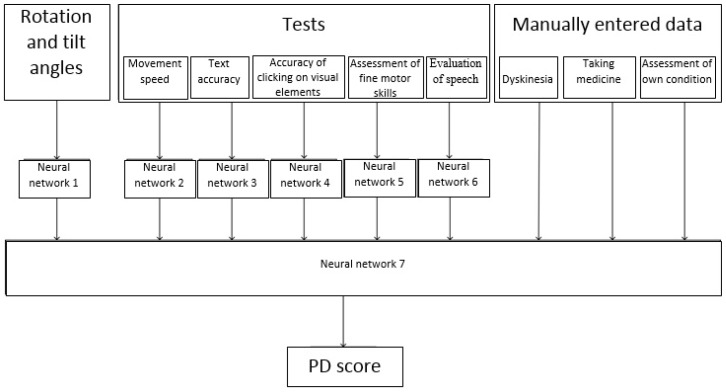
The process of diagnosing the severity of symptoms of Parkinson’s disease (PD).

To achieve this goal, it was necessary to build a number of neural networks to classify (evaluate) each of the parameters. Then, based on the obtained parameter estimates, the patient’s condition was classified using a neural network. This process is shown in Figure 2.

**Figure 3:**
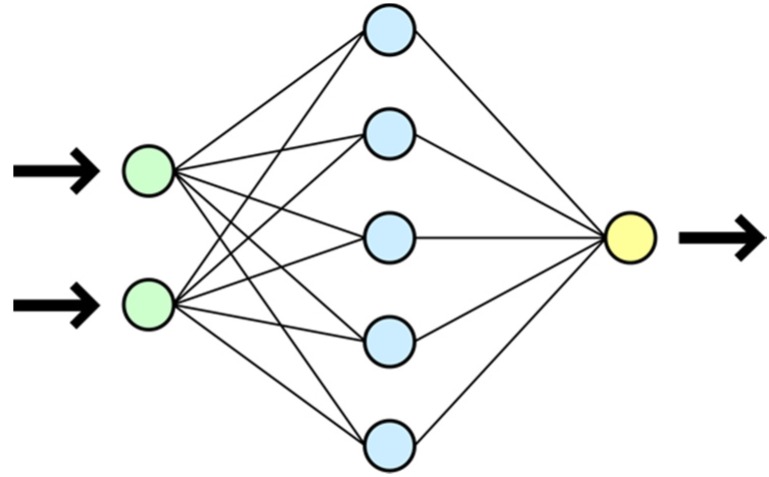
Scheme of a simple neural network. Green indicates input neurons, blue indicates hidden neurons, and yellow indicates output neuron.

A neural network is a sequence of neurons interconnected by synapses. A neuron is a computing unit that receives information, performs simple calculations on the information, and passes the information on further. They are divided into three main types of layers: input, hidden, and output. A synapse is a connection between two neurons. Synapses have one parameter called the weight. Thanks to the synapse, the input information changes form when it is transmitted from one neuron to another. The neural network diagram is shown in Figure 3.

The purpose of training a neural network is to obtain reliable results. Prediction is what the neural network returns after receiving input, for example, “given the number of drugs, the probability of tremor in a patient’s hands becomes lower is 60%”. Sometimes a neural network makes mistakes, but it can learn from them. If the predicted value is too high, it will reduce weight in order to get a lower predicted value next time, and vice versa.

It should be noted that in Figure 2 not all possible observable parameters are listed, however those for which data are currently being collected in the monitoring system for patients with PD are presented [22]. In the future, the range of observed parameters can be expanded, and the methods for evaluating each parameter changed.

One of the parameters used was the nature of the change in the angle of rotation and tilt of the phone while passing tests on it. Therefore, one of the tasks was used to assess changes of the angle of rotation and tilt of the mobile phone during the tests.

To solve this problem, it was necessary to:

- development of an application that allows the collection of training samples for a neural network using mobile devices;
- prepare and process data for a future neural network model;
- analyze of the possibility of using neural networks in order to classify the condition of patients with PD;
- select and test various variants of neural network architectures on the obtained sample;
- test the neural network in patients with PD;
- evaluate of the results of the neural network.

In the framework of this article, the process of evaluating changes in the values of the rotation angles and tilt of a mobile phone using a neural network is discussed in detail. For other parameters, similar studies are carried out.

## 4. The Choice of Neural Network Architecture, the Creation and Testing of the Network

One of the subtasks of this study was the choice of neural network architectures and the assessment of the possibility of using neural networks to classify the patient’s state according to the values of rotation angles and tilt angles of the mobile phone. Therefore, in this section, various options for neural network architectures are considered, the training procedure is described, and the results of network training are analyzed.

### 4.1. Input and Output Data of Neural Networks within One Subtask

Fragments of the input data of the values of the rotation angles and tilt of the mobile phone for the neural network to solve the problem of the classification of the patient’s state are presented in Table 2.

Obviously, each user can hold the phone in different ways and the angle of rotation and tilt of the phone will differ for different users. Therefore, to classify the condition of patients with PD, it is of interest not the absolute values of the angles, but the relative values and the frequency of their changes. The next step is to normalize the deviation values of each of the three parameters presented in Table 2 relative to the average value of the corresponding parameter. The result of the neural network is a number in the range from 0 to 1. A number is closer to 1 indicated more often the phone is in a less unbalanced state. However, the question remands whether this is it typical for patients with PD. Neural networks can help answer this question.

### 4.2. Neural Network Activation Functions

The activation function in neural networks determines the output signal depending on the set of input data. In hidden layers of neural networks, regardless of their architectures, the activation function is: *ReLU*(*x*) = max(0;*x*); in the output layer, this is the logistic activation function (sigmoid): $\sigma \left(x\right)=\frac{1}{1+e^{-x}}$. Sigmoid takes a real value as an input and displays a different value in the range from 0 to 1. It has the following properties: non-linear, continuously differentiable, monotonous, and has a fixed output range. The main disadvantage is vanishing gradients.

Unlike a sigmoid, ReLU is called a piecewise function as half of the output is linear (positive output) and the other half is non-linear. It does not suppress the neuron yield between 0 and 1, which helps with back propagation. ReLU provides the same benefits as Sigmoid, but with better performance and no vanishing gradient problem.

### 4.3. The Loss Function. Metrics. Neural Network Optimization Algorithm

When training a neural network, binary cross-entropy (BCE) [25] or standard deviation (MSE) can be used as a loss function:

$$BCE=-\hat{Y}\ln Y-\left(1-\hat{Y}\right)\ln\left(1-Y\right)$$

$$MSE=\frac{1}{n}\overset{n}{\underset{i=1}{\sum }}{\left(Y_{i}-\hat{Y_{i}}\right)}^{2}$$

The combined use of the standard deviation and the logistic activation function can lead to a situation where the gradients of the weights of the neural network turn to zero (this situation is called paralysis of the neural network), and further training becomes impossible. In this regard, binary cross-entropy is used as a loss function. The standard deviation is used as a metric of the quality of learning, since gradients are not calculated for the metrics.

When training a neural network, an optimization algorithm is implemented to minimizes the loss function. To train this neural network, the root mean square propagation (RMSProp) optimization algorithm is used [26]. This algorithm uses a moving average to normalize the gradient, which allows its output function to quickly converge to a given value.

### 4.4. Variants of Neural Network Architectures

As the initial data are a function of time, it is important to consider the previous values of these functions to obtain a reliable result. In this regard, to solve this problem, at least one recurrent layer must be present in the neural network, as layers of other types (fully connected and convolutional) cannot extract features from time dependencies.

Currently, two types of recurrent networks are used: Long Short-Term Memory (LSTM) and Gated Recurrent Unit (GRU).

The initial architecture of the neural network consists of four layers. The first is recurrent of 128 neurons, followed by fully connected layers of 64, 32, and one neuron. The number of neurons was selected by the heuristic method. The subsequent architectures created on the basis of the initial version will be obtained by adding layers, removing layers, and changing the type of layer without changing the number of neurons in the layer. Therefore, at the stage of training a neural network, the influence of its architecture (i.e., the number of layers and their types) on the learning outcome will be more pronounced in comparison with the influence of the number of neurons (on each layer) on the learning outcome.

As a possible improvement of the neural network, the introduction of a second recurrence layer was proposed. This increases the learning speed and accuracy of the neural network by reducing the time it takes to calculate the results of classifying the status of patients with PD based on the data provided.

This change of architecture leads to an improvement in the case if the initial data sequences form more complex dependencies that cannot be detected with a single layer.

**Figure 4:**
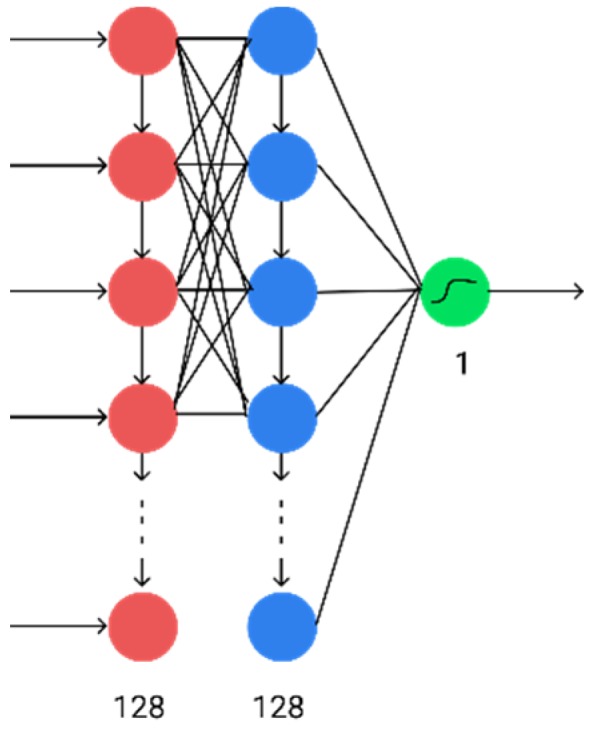
A fully recurrent version of a neural network. Blue and red are the first and second recurrent layers, green is the result.

Next, hidden fully connected layers were removed. In this case, a completely recurrent neural network was obtained, the architecture of which is shown in Figure 4. In this case, there was an improvement in the performance of computing the results (by reducing the number of layers) with only a slight deterioration in accuracy.

For original data sets, it is advisable to choose an architecture with three hidden layers, since an advantageous compromise is achieved between system performance and accuracy.

### 4.5. Neural Network Training

**Figure 5:**
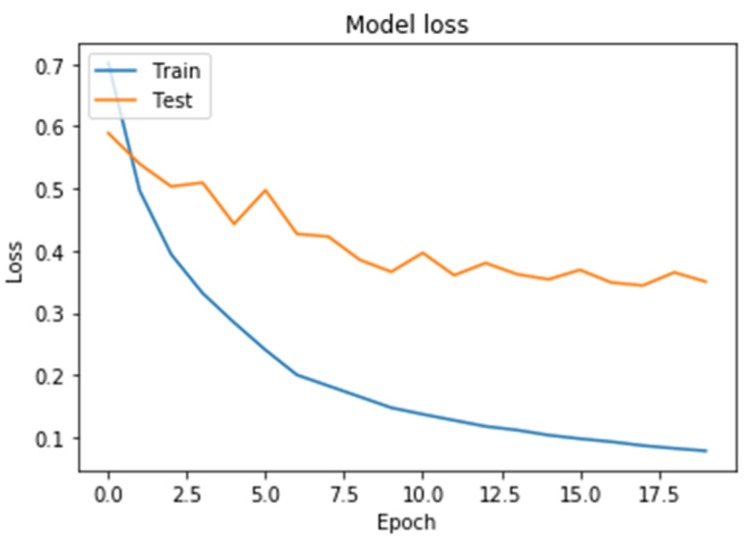
The results of training a neural network with one recurrent layer.

**Figure 6:**
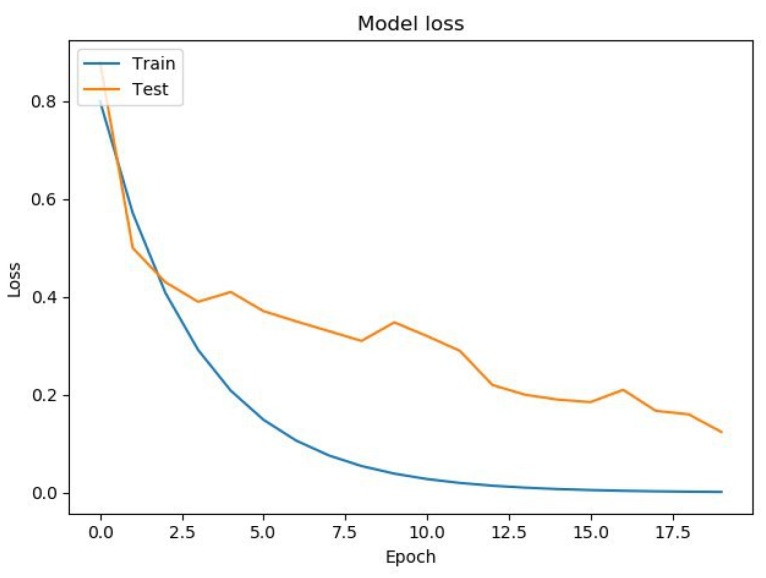
The results of training a neural network with two recurrent layers.

**Figure 7:**
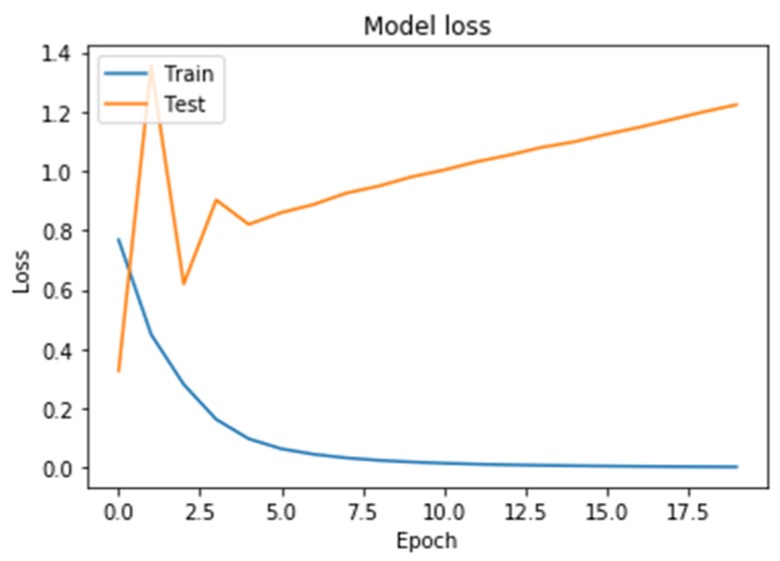
The results of training neural network with a fully recurrent architecture (gated recurrent unit; GRU).

**Figure 8:**
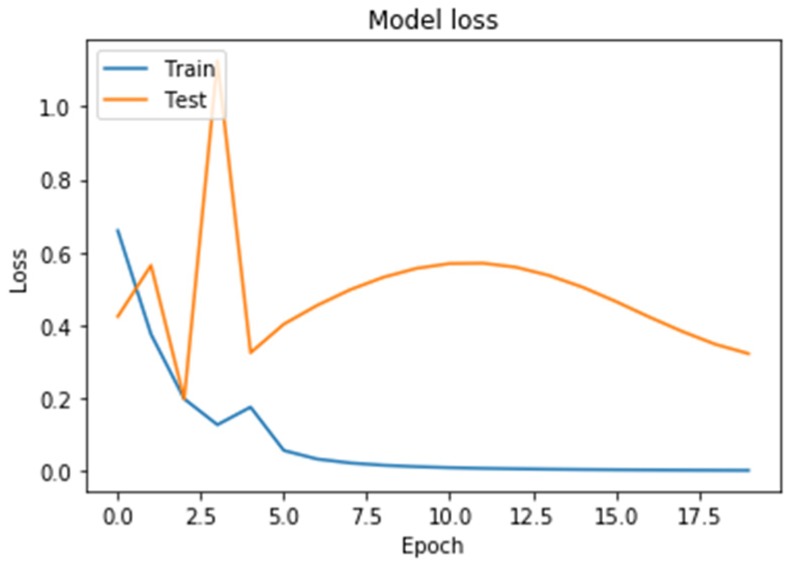
The results of neural network training with fully recurrent architecture (Long Short-Term Memory; LSTM).

Learning outcomes are shown in Figure 5, Figure 6, Figure 7 and Figure 8. On the horizontal axis is the serial number of the training epoch, and on the vertical axis is the standard deviation of the result of neural network calculations from the corresponding value in the validation sample. The blue line indicates the loss function in the training set, and the orange line in the test set. As the returned values do not have units, the standard deviation also does not have units.

Figure 5 shows a graph of the loss function when training a neural network with one recurrent layer and two fully connected layers. It can be seen from the Figure 5 that both the learning error and the testing error monotonously decreased. This means that the neural network is capable of generalizing the source data.

Figure 6 shows a graph of the loss function in training a neural network with two recurrent and two fully connected layers. Learning and testing errors decreased, and the value of the testing error for this architecture was less than for a neural network with one recurrent layer, i.e., it generalized data better.

Figure 7 shows a graph of the loss function in training a neural network with two GRU recurrent layers. As a result of training a neural network using this architecture, it led to an increase in the graph of the testing error function from the beginning of training.

Figure 8 shows a graph of the loss function in training a neural network with two recurrent LSTM layers. The behavior of the test error curve did not correspond to the state of retraining. The value of the loss function was higher than that of a neural network with two recurrent layers.

Table 3 presents the results of further training of all the neural networks described above.

An analysis of the results of training neural networks of various architectures shows that an architecture with two recurrent and two fully connected layers had greater accuracy compared to other options. Thus, a neural network with two recurrent and two fully connected layers best generalizes the data and is suitable for further training.

### 4.6. Neural Network Implementation

TensorFlow and Keras libraries were used to build and train neural networks. They provide implementations of various types of recurrence layers, including versions that use GPU computing. In order to train neural networks in this study, we used a back propagation method, an implementation of which is also provided in Keras.

Neural network training was implemented using the following code:

          *// Create a neural network of sequential execution (each layer is associated with only one subsequent)*          *model = Sequential()*          *// Create GRU recurrence layers with 128 neurons*          *model.add(tf.keras.layers.LSTM(128), return_sequences = True)*          *model.add(tf.keras.layers.LSTM(128))*          *// Create fully connected layers with 64 and 32 neurons and a ReLU activation function*          *model.add(Dense(64, activation = ‘relu’))*          *model.add(Dense(32, activation = ‘relu’))*          *// Create a fully connected layer with 1 neuron and logistic activation function*          *model.add(Dense(1, activation = ‘sigmoid’))*          *// Compilation of a neural network with binary cross-entropy as a function of losses, standard deviation as a metric and RMSProp gradient descent optimization algorithm*          *model.compile(loss = ‘binary_crossentropy’, optimizer = ‘rmsprop’, metrics = [‘mse’])*          *// Start the training procedure. 20 epochs will be passed, 20% of the initial data are used as a validation sample*          *history = model.fit(X_norm, y, epochs = 20, validation_split = 0.2)*

Using libraries significantly speeds up the development time of neural networks, allowing to quickly implement and test various architectures that eliminate the need to write low-level and template code.

## 5. Future Perspective

Currently, studies are being conducted on the application of the results of neural networks constructed according to tests and on changes in the rotation angles and tilt angles of the telephone to build a common neural network.

The results of a general neural network in the monitoring system for the condition of patients with PD will be used to track the dynamics of changes in the patient’s condition and the effect of drugs. Further scientific research regarding the determination of the parameters that are available for measurements using mobile phones that most characterize the patient’s condition will be useful.

## 6. Limitations

The main limitation of this work is that preliminary assessments of the conditions by the patients themselves were used for the training and evaluation of constructed neural network models. However, in the early stages of PD, data from the sensors of patients’ phones cannot yet be clearly distinguishable from data on the state of a healthy person. This is a new problem for our study, and has not been solved in the world. We hope that if we can collect much more data, we will get closer to solving it. Much more data is needed both in volume and in the number of parameters in order to more accurately determine the stability of tests based on machine learning and smartphones to these mixed factors.

## 7. Conclusions

The availability of mobile phones to monitor patients with PD can have a profound impact on clinical practice, giving doctors access to long-term data. These additional data can help doctors obtain a more complete and objective understanding of the symptoms and fluctuations of their patients’ symptoms and, therefore, will allow for more accurate diagnoses and treatment regimens.

We are creating a system of information and technological support for research on Parkinson’s disease, taking into account the collection and processing of data of a large volume. Currently, in this system we have already created most of the modules, such as collecting data on the nature of the use of the phone and automatically filling out the patient’s diary according to the data entered by the patient on the phone. Partially, we described this in Section 3 of this article. A more complete description can be found in one of our previous articles [22]. Using this system, data is sent daily to the doctor’s computer. This eliminates the disadvantage of the lack of constant communication with the specialist, which we discussed in Section 2. The doctor has the opportunity to see a continuous graph of the dynamics of the patient’s condition. We have shown that it is possible to use neural networks, which can make filling out a patient’s diary without his participation and make this filling out better.

As a result of this work, it was determined that the neural network is able to summarize data on the rotation angles and tilt angles of the mobile phone, in order to classify the condition of patients with PD. In this paper, we considered and carried out a comparative analysis of various architectures for building neural networks. An architecture of a neural network with two recurrent layers was chosen, at which an acceptable level of accuracy in classifying the state of a patient with PD was achieved.

Python neural networks were built using the TensorFlow and Keras libraries. It was established that the use of LSTM blocks instead of GRU leads to greater accuracy of the neural network. Future research will focus on the construction of neural networks for more parameters and a larger sample of the source data, which will lead to the training of the neural network so that it will return even more accurate diagnosis results.

## Figures and Tables

**Table 1:** Parameters obtained using tests on mobile phones.

Parameter	Parameter Description	Value Example	Unit
Text erased	Number of characters deleted	13	-
Text time	Time for writing text in a special test	139,763	ms
Levenstein Distance	Metric measuring the difference between two sequences of characters	5	-
Miss clicks	The number of misses when clicking buttons in the application	4	-
Miss clicks distance	The distance between the center of the nearest button and the center of the finger touch the phone screen	2.357022	dp
Azimuth	The longitudinal axis of the coordinate system	70.29761	degree
Pitch	The transverse axis of the coordinate system	−80.30805	degree
Roll	The vertical axis of the coordinate system	13.761927	degree
Tapping left count	The number of touches by the index finger of the left hand of the button on the screen in 1 min in a special test	47	-
Tapping right count	The number of touches with the index finger of the right hand of the button on the screen for 1 min in a special test	52	-
Dyskinesia	The presence of dyskinesia	1	-
Pill	The number of medications taken	4	-
State	Subjective assessment of the patient’s condition (0 - poor, 0.5 - uncertain, 1 - good)	1	-
Voice volume	Voice volume	44	decibels
Voice pause	The number of pauses between words	3	-
Voice count pause	The pause time between words	4794	ms
Velocity	The speed of the phone during its active use	1.342	ms

dp or dip (density-independent pixels) is an abstract unit of measurement that allows applications to look the same on different screens and resolutions, ms is milliseconds.

**Table 2:** An example of input data.

Azimuth	Pitch	Roll
70.29761	−80.30805	13.761927
80.7381	88.84665	−0.4941308
103.91639	89.57664	−23.674112
120.00478	89.701294	−39.76287
136.86708	89.748215	59.555275

Data in the Table 2 is sorted by azimuth. But, the azimuth values in the first and last line are not necessarily the minimum and maximum values. Such numbers were obtained in this study. In other tests, they may be different.

**Table 3:** The results of training of neural networks of various architectures.

Architecture	The Number of Epoch before Retraining	Loss Function (BCE)	Accuracy
1 GRU + 2 fully connected	29	0.031161	0.817
2 GRU + 2 fully connected	32	0.029795	0.88951
2 LSTM	26	0.03809	0.8528

LSTM is Data in the Long Short-Term Memory, GRU is Gated Recurrent Unit, BCE is binary cross-entropy.
